# Epidemiological risk factors associated with primary infection by Epstein–Barr virus in HIV-1-positive subjects in the Brazilian Amazon region

**DOI:** 10.1038/s41598-021-97707-4

**Published:** 2021-09-16

**Authors:** Leonn Mendes Soares Pereira, Eliane dos Santos França, Iran Barros Costa, Igor Tenório Lima, Amaury Bentes Cunha Freire, Francisco Lúzio de Paula Ramos, Talita Antonia Furtado Monteiro, Olinda Macedo, Rita Catarina Medeiros Sousa, Felipe Bonfim Freitas, Igor Brasil Costa, Antonio Carlos Rosário Vallinoto

**Affiliations:** 1 Laboratory of Virology, Institute of Biological Sciences, Federal University of Pará, Belém, Pará Brazil; 2 Epstein-Barr Virus Laboratory, Virology Section, Evandro Chagas Institute, Ananindeua, Pará Brazil; 3 Department of Epidemiology and Surveillance, Evandro Chagas Institute, Ananindeua, Pará Brazil; 4 Laboratory of Retroviruses, Evandro Chagas Institute, Virology Section, Ananindeua, Pará Brazil; 5 School of Medicine, Federal University of Pará, Belém, Pará Brazil; 6 Graduate Program in Biology of Infectious and Parasitic Agents, Institute of Biological Sciences, Federal University of Pará, Belém, Pará Brazil

**Keywords:** Retrovirus, Viral epidemiology

## Abstract

To identify the prevalence and risk factors for primary Epstein–Barr virus (EBV) infection in human immunodeficiency virus (HIV)-1-positive adult treatment-naïve patients between January 2018 and December 2019 in a state of the Brazilian Amazon region. A total of 268 HIV-1 positive patients and 65 blood donors participated in the study. Epidemiological data were obtained from medical records and through a designed questionnaire. EBV infection was screened by the semiquantitative detection of anti-viral capsid antigen (VCA) EBV IgM and IgG, followed by molecular detection of the EBNA-3C gene. The plasma viral loads of HIV-1 and EBV were quantified using a commercial kit. The prevalence of primary coinfection was 7.12%. The associated risk factors were education level, family income, history of illicit drug use and sexually transmitted infections, homosexual contact and condom nonuse. Approximately 58.5% had late initiation of highly active antiretroviral therapy, which influenced the risk of HIV-EBV 1/2 multiple infection (odds ratio (OR): 4.76; 95% CI 1.51–15.04) and symptom development (p = 0.004). HIV viral load was associated with patient age (OR: 2.04; 95% CI 2.01–2.07; p = 0.026) and duration of illicit drug use (OR: 1.57; 95% CI 1.12–2.22; p = 0.0548). EBV viral load was associated with younger age (OR: 0.82; 95% CI 0.79–1.03; p = 0.0579). The replication of both viruses was associated with symptom development (HIV = OR: 2.06; 95% CI 1.22–3.50; p = 0.0073; EBV = OR: 8.81; 95% CI 1–10; p = 0.0447). The prevalence of HIV/EBV coinfection was lower than that observed in other studies, and social vulnerability and promiscuous sexual behavior were associated risk factors. A long time of HIV-1 infection, without therapy, influenced the risk of coinfection and disease progression. The viral loads of both viruses may be associated with some epidemiological aspects of the population.

## Introduction

The Epstein–Barr virus (EBV), also known as human gammaherpesvirus 4 (HHV4), belongs to the family *Herpesviridae*, genus *Lymphocryptovirus*^1,2^. Two EBV genotypes are classically known, EBV-1 and EBV-2, whose genetic differences lie in the sequence encoding the viral nuclear antigens (EBNA2, -3A, -3B and -3C)^3,4^. Both genotypes have a cosmopolitan distribution, although EBV-1 is more prevalent, especially in Western countries and Asia^5^, while EBV-2 is more prevalent in Central African countries^6^.

In developed countries, EBV infection is common in adolescence, ranging from 0 to 70% in childhood and reaching more than 90% in adulthood; in the latter age group, primary infection is clinically symptomatic^7–9^. In developing countries, primary EBV infection occurs in early childhood and is usually asymptomatic, reaching 97% seroprevalence in adulthood in heterogeneous populations, a value that is similar at the global scale^10,11^. In these countries, there is an alert for seroprevalence in children and adolescents with low socioeconomic status^12^ and for the predominance of infection in individuals with suspected coinfection with human immunodeficiency virus (HIV)-1^13^.

Because it is a precursor of lymphatic neoplasms, EBV is identified as one of the factors that contributes to the morbidity of patients infected with HIV-1^14–16^. Coinfection is related to progressive dysfunction events and impaired immune surveillance caused by HIV that favor persistent pathogens^17^. This factor becomes critical when the infection progresses to AIDS, in which severe immunosuppression results in clinical signs and symptoms related to malignant neoplasms^18,19^.

Although most studies focus on the pathological basis of viral carcinogenesis, it is still necessary to understand aspects of primary EBV infection in the context of coinfection. In this sense, EBV coinfection has been studied in HIV-1-seropositive children, in whom EBV DNA was isolated from the oral mucosa^20^ and blood tissue^21,22^. It is assumed that due to the high incidence of EBV infection during the first 5 years of life, maternal transmission is an important route in maintaining coinfection^21,23^. Additionally, microbial translocation and persistent immune activation induced by HIV are factors that may influence EBV replication and the expansion of infected B cells^22^.

In adults, exposure through unprotected sex can favor HIV/EBV coinfection^24^. However, there is controversy regarding the detection of EBV in different HIV-1 infection profiles^25^. A recent study reports higher than 68% primary EBV infection in patients with HIV not on highly active antiretroviral therapy (HAART)^26^, similar to the rate observed in patients on the HAART regimen^27^. These findings indicate the complexity of the relationship between viruses that can be attributed to intrinsic factors of coinfection, regardless of the therapeutic status of the patient^28^.

There are few scientific studies on the epidemiology of HIV/EBV coinfection in the Brazilian Amazon and northern region of the country. Most research is focused on the carcinogenic effects of viral interactions^29,30^. To date, we have only found 1 study, by Jacome-Santos et al., that reported the overall prevalence of coinfection by strictly analyzing the presence of EBV in the oral mucosa of people living with HIV (PLHIV)^31^. Thus, the aim of the present study was to describe the prevalence of primary EBV infection in HIV-positive adult therapy-naïve and to identify the epidemiological characteristics associated with this profile.

## Materials and methods

### Sample characterization and ethical aspects

This was an observational, cross-sectional and analytical study conducted in mutual collaboration between the Laboratory of Virology of the Federal University of Pará (LABVIR-UFPA), the Evandro Chagas Institute (IEC) and the Hemotherapy and Hematology Foundation of Pará (HEMOPA), with the selection of participants who regularly resided in municipalities of the state of Pará, Brazil, from January 2018 to December 2019, originating from the Center for Health Care in Acquired Infectious Diseases (CASA DIA), HEMOPA and IEC.

All participants were clinically evaluated at the respective referral centers and subjected to complementary investigation via serological and molecular biology tests, based on which the participants were categorized as follows: 249 HIV-1 monoinfected patients (p24+; anti-HIV-1 IgG+); 19 HIV-1-positive patients with primary EBV infection (p24+; anti-HIV-1 IgG+; anti-viral capsid antigen (VCA)-EBV IgM+; anti-VCA-EBV IgG−; confirmation by molecular biology); and 65 uninfected individuals (volunteer blood donors, based on a screening panel of blood banks defined by ministerial ordinance^32^).

Epidemiological data, such as sex, age, place of residence, and clinical anamnesis of patients, were obtained from clinical records and an epidemiological questionnaire administered at the time of care. In compliance with resolutions 466/2012 and 347/05 of the Brazilian National Health Council, which establishes guidelines and regulatory standards for research involving human beings, the project proposal was submitted for ethical review and approved by the Human Research Ethics Committee of IEC (Protocol n. 3.121.265; CAAE n. 73927717.3.0000.0019). All participants were informed about the study objectives and signed an informed consent form. The collected biological samples were stored in a biorepository until use.

Participants included in the study were 18 years of age or older, of both sexes, carriers of HIV-1 and/or EBV, antiretroviral therapy-naïve and signed the informed consent form. Participants who did not meet the inclusion criteria or who were on antiviral therapy before sample collection were excluded.

### Collection, extraction and confirmatory methods

Five milliliters of peripheral blood was collected into vacuum tubes containing EDTA as an anticoagulant. DNA was extracted from whole blood following the protocol provided with the QiaAmp DNA Mini Kit (Qiagen, Germany).

HIV-1 infection was screened by qualitative and simultaneous detection of p24 antigen and anti-HIV-1 and anti-HIV-2 IgG antibodies by enzyme immunoassay (Murex HIV Ag/Ab Combination, DiaSorin, UK); serological confirmation was performed using an Immunoblot rapid DPP HIV-1/2 kit (Bio-Manguinhos, FIOCRUZ) following the manufacturer's recommendations. The samples from CASA DIA did not require complementary tests for the diagnosis of HIV-1 because the institution has its own screening panel, to which patients enrolled in the institution are subjected.

Infection by EBV was screened by semiquantitative detection of anti-EBV IgM and IgG antibodies by enzyme immunoassay (Ridascreen EBV VCA R-Biopharm, Germany) following the manufacturer's recommendations. The identification of EBV genotypes was performed by nested PCR, targeting the EBNA-3C gene, using the primers described by Lorenzetti et al.^33^ ((1st round) (F: 5′-AGATGGTGAGCCTGACGTG-3′/R: 5′-GCATCCTTCAAAACCTCAGC-3′)) and by Sample et al.^3^ ((2nd round) (F: 5′-AGAAGGGGAGCGGTGTGTTGT-3′/R: 5′-GGCTGTTTTTGACGTCGGC-3′)) and following the recommended conditions: primers (10 pmol/µL); MgCl_2_ (50 mM); dNTP (10 mM); and Taq (5U/µL); 1st round cycles—1 cycle of 95° C/3′, 20 cycles of (94 °C/45″; 56 °C/45″; 72 °C/45″), 1 cycle of 72 °C/7′; and 2nd round cycles—1 cycle of 95° C/3′; 35 cycles of (94 °C/45″; 56 °C/45″; 72 °C/45″); 1 cycle of 72 °C/7′. The presence of a 153-bp fragment was considered positive for EBV-1, and the presence of a 246-bp fragment was considered positive for EBV-2. The positive EBV-1 and EBV-2 controls were isolated from the B95 and P3HP1 lymphoid cell lines, respectively.

### Quantification of HIV-1 and EBV viral load

The HIV-1 plasma viral load was quantified by real-time PCR using an Abbott mSample Preparation System RNA extraction kit and an Abbott Real-Time HIV-1 amplification reagent kit (Abbott, Chicago, Illinois, USA) following the manufacturer’s recommendations.

The EBV viral load was quantified by real-time PCR following the protocol provided with the XGEN Master EBV kit (Mobius Life Science, Pinhais, Paraná, Brazil). Plasma samples were used to quantify the viral load.

### Statistical analysis

The epidemiological data were compared among the study groups using the G test. Fisher’s exact test was applied exclusively in comparisons whose data were arranged in 2 × 2 contingency tables.

For significant comparisons, the degree of dependence between the epidemiological data and the study groups was calculated using simple logistic regression for 1 variable and multiple logistic regression for all variables, in which epidemiological data were included as independent variables and the presence or absence of infections as dependent variables. Due to the statistical similarity between the epidemiological profile of HIV-1 monoinfected and HIV-1/EBV coinfected patients, we assumed the presence of monoinfection or coinfection as success (1) and the absence of both as nonsuccess (0). The epidemiological factors that maintained statistical significance in the multiple regression were considered risk factors for monoinfection or coinfection.

We also calculated the dependence of the increase in viral load on the epidemiological and behavioral variables of patients. In this context, we calculated the mean viral load values and classified them, for HIV, into low (0), when between undetectable and up to 10,000 RNA copies and high (1), when greater than 10,000 copies, and, for EBV, into low (0), when between undetectable and up to 45 DNA copies, and high (1), when higher than 45 copies. The quantitative epidemiological variables were included in the function as nonbinary data, and exclusively for the length of illicit drug use, we discarded patients with noncontinuous sporadic use and grouped the remaining time of use into less than 1 year, 1 to 5 years, or greater than 5 years.

The G test was applied in other analyses of categorical data, such as in the comparison of the profile of sexually transmitted infections (STIs); behavioral changes according to the HIV-1 infection time (since diagnosis) and symptomatology. We used curve fitting to determine the frequency distribution model of cases of monoinfection and coinfection according to the collection time.

All statistical analyses were performed using the programs GraphPad Prism 3.03 (San Diego, CA, USA) and Bioestat 5.0^34^.

### Ethics approval and consent to participate

All methods and experimental protocols were carried out in accordance with regulations 466/2012 and 347/05 of the Brazilian National Health Council and were approved by the Human Research Ethics Committee of Instituto Evandro Chagas (Protocol n. 3.121.265; CAAE n. 73927717.3.0000.0019). All participants were informed about the study objectives and signed an informed consent form. The collected biological samples were stored in a biorepository until use.

## Results

**Figure 1:**
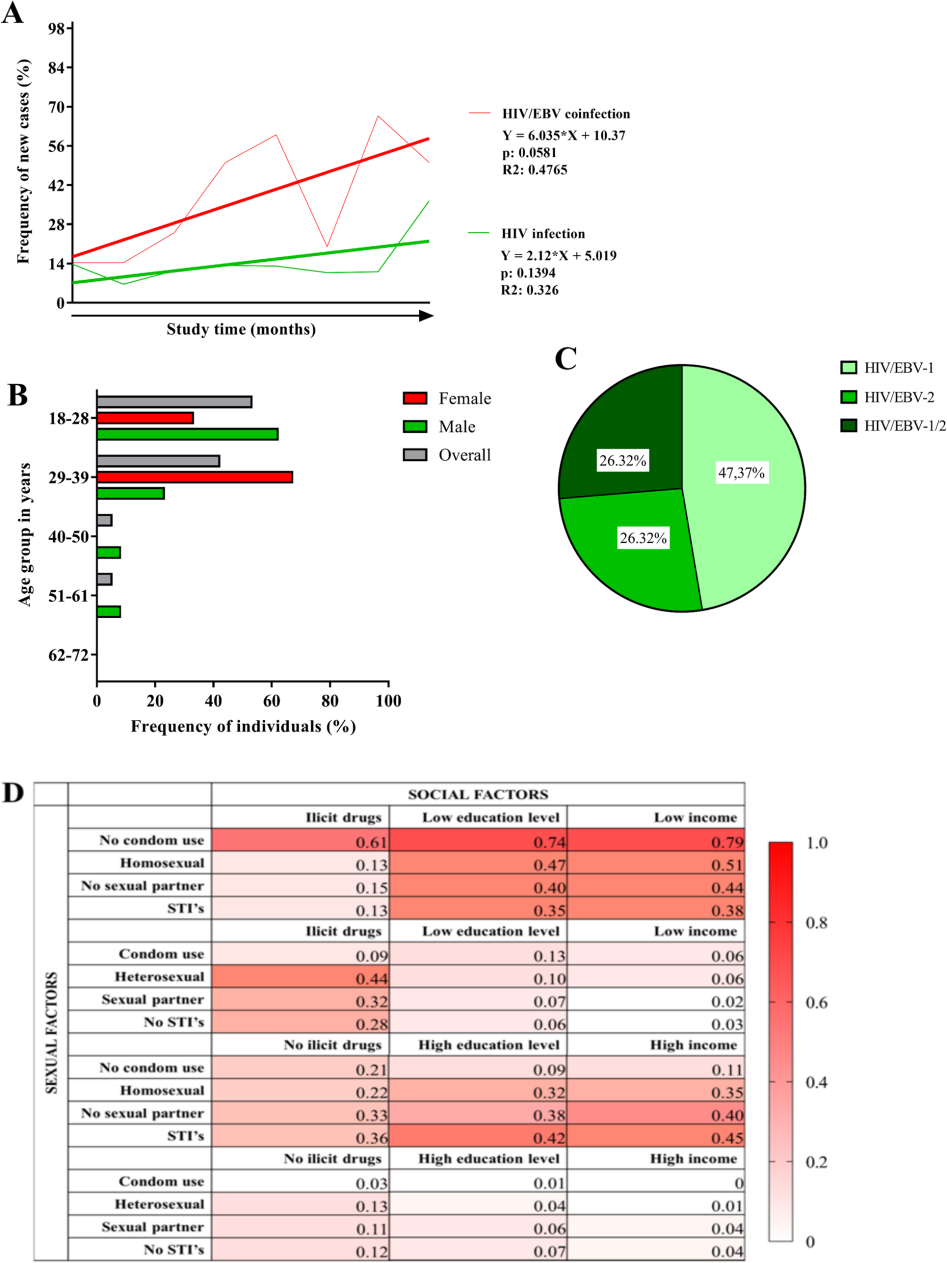
Frequency of cases and degree of exposure: (**A**) Frequency of new cases of HAART-free HIV-1 patients coinfected with HIV/EBV in the period between January 2018 and December 2019. (**B**) Prevalence of cases of HIV/EBV coinfection stratified based on age and sex. (**C**) Prevalence of EBV genotypes among HIV/EBV coinfected patients. (**D**) Potential risk/exposure to HIV-1 monoinfection or co-infection. The color gradient was proposed based on data on the frequency of individuals screened according to the intersection of social and sexual factors.

We detected primary EBV infection in only 19 of the 268 HIV+ patients evaluated (7.12%) (Table 1) and did not detect the presence of primary EBV infection in blood donor samples. The frequency of coinfection cases increased linearly throughout the study, approximately sixfold per month of collection (p = 0.0581; R^2^: 0.476), although the increase in HIV-1 monoinfection was slight (p = 0.1394; R^2^: 0.326) (Fig. 1A). The prevalence of coinfection cases was higher in the group of patients 18 to 28 years of age, specifically in men; the number of infected women stood out only in the group of patients 29 to 39 years of age (Fig. 1B). The EBV-1 genotype was the most prevalent among the coinfected patients (47.37%); EBV-2 and multiple infection by the 2 genotypes (EBV-1/2) occurred at the same frequency (26.32%) (Fig. 1C).

**Table 1:** Description of socio-epidemiological aspects and risk factors associated with HIV/EBV co-infection.

Groups	HIV n: 249	HIV/EBV n: 19 (7.12)	Not infected n: 65	Simple logistic regression			Multiple logistic regression		
Factors				p-values	OR	IC (95%)	p-values	OR	IC (95%)
**Sex**									
Female	49 (19.7)	6 (31.6)	17 (26.2)	NS*					
Male	200 (80.3)	13 (68.4)	48 (73.8)						
**Age**									
18–28	126 (50.6)	10 (52.6)	15 (23.1)	0.0078^#^	1.57	1.20–2.05	NS		
29–39	66 (26.5)	7 (36.8)	30 (46.2)						
40–50	40 (16.1)	1 (05.3)	10 (15.4)						
51–61	13 (05.2)	1 (05.3)	9 (13.8)						
62–72	4 (01.2)	0	1 (01.5)						
**Education**									
Not literate	1 (00.4)	0	0	< 0.0001^#^	0.21	0.13–0.32	0.0028	0.06	0.01–0.04
Literate	34 (13.7)	2 (10.5)	0						
Elementary school 1	46 (18.5)	6 (31.6)	4 (06.6)						
Elementary school 2	131 (52.6)	9 (47.4)	21 (32.3)						
High school	36 (14.5)	2 (10.5)	26 (40.0)						
University education	1 (00.4)	0	14 (21.5)						
**Family income**									
Unknown	9 (03.6)	1 (05.3)	1 (01.5)	0.0418^#^	0.48	0.33–0.70	0.0002	0.45	0.27 – 0.67
(< 1) salário	41 (16.5)	8 (42.1)	9 (13.8)						
(1–3) wage	180 (72.3)	10 (52.6)	37 (56.9)						
(4–6) wages	15 (06.0)	0	12 (18.5)						
(7–10) wages	2 (00.8)	0	3 (04.6)						
(> 10) wages	3 (01.2)	0	3 (04.6)						
**Cigarette use**									
Historic									
No	113 (45.4)	10 (52.6)	46 (70.8)	0.0013*	2.95	1.64–5.30	NS		
Yes	136 (54.6)	9 (47.4)	19 (29.2)						
Current usage									
No	101 (74.3)	6 (66.7)	17 (89.5)	NS*					
Yes	35 (25.7)	3 (33.3)	2 (15.5)						
**Alcohol consumption**									
Historic									
No	31 (12.4)	2 (10.5)	9 (13.8)	NS*					
Yes	218 (87.6)	17 (89.5)	56 (86.2)						
Present use									
No	109 (50.0)	10 (58.8)	13 (23.2)	0.0006*	0.29	0.15–0.58	NS		
Yes	109 (50.0)	7 (41.2)	43 (76.8)						
**Illicit drug use**									
Historic									
No	177 (71.1)	14 (73.7)	63 (96.9)	< 0.0001*	24.53	3.34–180.14	0.0367	13.74	1.18–160.46
Yes	72 (28.9)	5 (26.3)	2 (03.1)						
Present use									
No	70 (97.2)	5 (100.0)	2 (100.0)	NS*					
Yes	2 (02.8)	0	0						
**Routes of administration**									
Oral	5 (06.9)	0	0	NS^#^					
Inhale	65 (90.3)	5 (100.0)	2 (100.0)						
Injectables	0	0	0						
**Family history of cancer**									
No	147 (59.0)	12 (63.2)	34 (52.3)	NS*					
Yes	101 (41.0)	7 (36.8)	31 (47.8)						
**Sexual orientation**									
Heterosexual	106 (42.6)	9 (47.4)	63 (97.0)	< 0.0001^#^	10.87	4.32–27.34	0.0008	06.80	2.23–20.75
Homosexual	107 (43.0)	7 (36.8)	1 (01.5)						
Bisexual	36 (14.5)	3 (15.9)	1 (01.5)						
**Active sex life**									
Yes	160 (64.3)	11 (57.9)	54 (83.1)	0.0110*	0.36	0.18–0.72	NS		
No	89 (35.7)	8 (42.1)	11 (16.9)						
**First sexual contact**									
No relationships	0	0	1 (01.6)	NS^#^					
Do not remember	1 (00.4)	0	0						
< 9 years	0	0	0						
10–15 years	115 (46.2)	13 (68.4)	16 (24.6)						
16–20 years	118 (47.4)	5 (26.3)	45 (69.2)						
21–25 years	14 (05.6)	1 (05.3)	3 (04.6)						
> 25 years	1 (00.4)	0	0						
**Steady partner**									
No	121 (48.6)	5 (26.3)	9 (13.8)	< 0.0001*	4.81	2.25–10.25	0.0349	2.87	1.00–2.92
Yes	128 (51.4)	14 (73.7)	56 (86.2)						
**Relations with sex workers**									
No	191 (76.7)	16 (84.2)	55 (84.6)	NS*					
Yes	58 (23.3)	3 (15.8)	10 (15.4)						
**Condom use**									
Occasionally	143 (57.4)	9 (47.4)	17 (27.0)	< 0.0001	1.46	1.06–2.01	0.0034	6.23	1.83–21.19
Never	30 (12.0)	4 (21.1)	25 (39.7)						
Always	76 (30.6)	6 (31.6)	21 (33.3)						
**History of STIs**									
No	148 (59.4)	8 (42.1)	62 (95.4)	< 0.0001*	14.05	4.29–45.95	0.0024	13.1	2.50–69.06
Yes	101 (40.6)	11 (57.9)	3 (04.6)						

NS: not significant; *Fisher's exact test; ^#^G Test.

The epidemiological profiles of patients monoinfected with HIV-1 and coinfected with HIV/EBV were statistically similar; therefore, we grouped them for comparison with the noninfected group. Evaluating the variables individually, we observed that age was a risk factor for monoinfection or coinfection, being prevalent in the groups of young patients between 18 and 39 years of age (p = 0.0078; odds ratio (OR): 1.57; 95% CI 1.20–2.05). Only a history of cigarette use (p = 0.0013; OR: 2.95; 95% CI 1.64–5.30) and illicit drug use (p < 0.0001; OR: 24.53; 95% CI 3.34–180.14) were considered risk factors; present use was not relevant because most individuals claimed to have ceased these practices. Most aspects related to sexual practice and sexuality were associated with monoinfection or coinfection: history of STIs (p < 0.0001; OR: 14.05; 95% CI 4.29–45.95) and homosexual contact (p < 0.0001; OR: 10.87; 95% CI 4.32–27.34) had the highest probability ratios, followed by lack of a steady partner (p < 0.0001; OR: 4.81; 95% CI 2.25–10.25) and occasional condom use (p < 0.0001; OR: 1.46; 95% CI 1.06–2.01). An active sex life was associated with the noninfected group (p = 0.0110; OR: 0.36; 95% CI 0.18–0.72), whereas biological sex, history of sexual contact with sex workers and first sexual contact were not associated with infected patients (Table 1).

In contrast, higher education levels and higher family income were considered protective factors against monoinfection and coinfection, with the majority of noninfected individuals having completed secondary education or higher (p < 0.0001; OR: 0.21; 95% CI 0.13–0.32) and showing a greater representation of incomes ranging from 4 to greater than 10 times the minimum wage (p = 0.0418; OR: 0.48; 95% CI 0.33–0.70). Notably, current alcohol consumption was more strongly associated with noninfected patients (p = 0.0006; OR: 0.29; 95% CI 0.15–0.58). A family history of cancer and route of illicit drug administration were not associated with infected patients; most individuals reported no cases of cancer in the family, and for patients with a history of illicit drug use, most ingested them (Table 1).

We defined the risk factors for monoinfection or coinfection based on the multiple logistic regression results. In this context, education level, family income, history of illicit drug use, history of STIs, sexual orientation, and presence of a steady partner and condom nonuse remained significant. History of illicit drug use was the strongest risk factor for monoinfection or coinfection (p = 0.0367; OR: 13.74; 95% CI 1.18–160.46), and high family income was the strongest protective factor (p = 0.0002; OR: 0.45; 95% CI 0.27–0.67) (Table 1).

We generated a risk/potential matrix of exposure to monoinfection or co-infection that compiled risk factors categorized as sexual or social; the color gradient was based on the frequency of cases screened by the intersection of social and sexual factors (Fig. 1D). The level of risk/exposure was higher in individuals who did not use condoms, used illicit drugs, had low education and low income. High-income individuals had a low level of risk/exposure only when related to non-promiscuous sexual behaviors. Heterosexual orientation was considered a low risk/exposure factor only when associated with not using illicit drugs or high education and high income.

We also investigated the time of HIV-1 infection since diagnosis in the monoinfected and coinfected groups. A considerable portion of the patients (48.42%) lived for less than 1 month with HIV-1 and sought specific care immediately after diagnosis; however, approximately 51.58% of the patients lived between 1 and more than 12 months with the virus and without the use of HAART. When asked, patients with a longer time since diagnosis reported initial skepticism of the infection. Their point of view changed with the onset of symptoms, including through advice from third parties (data not shown).

**Figure 2:**
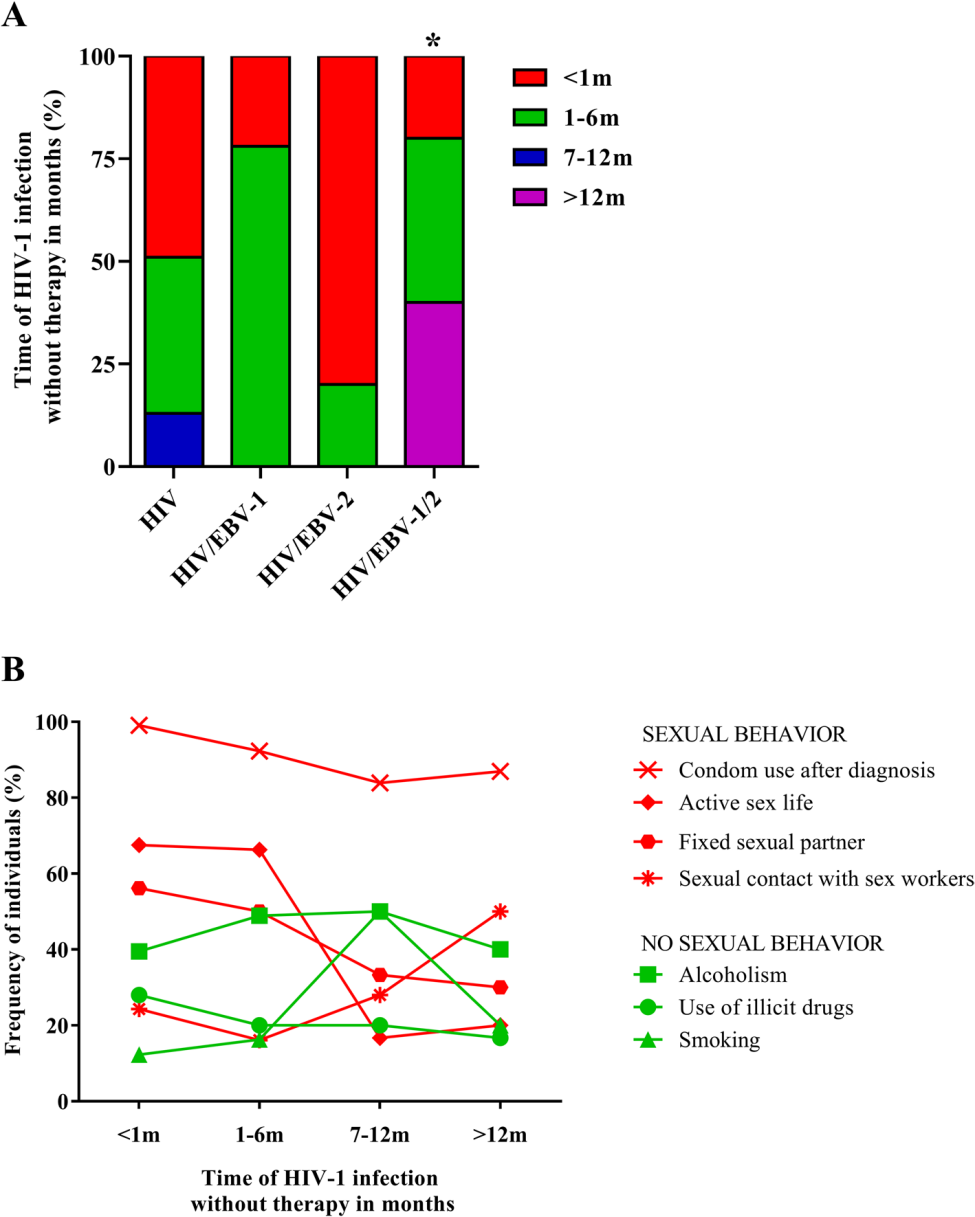
Late initiation of treatment for HIV-1 infection: (**A**) Frequency for initiation of HIV-1 infection treatment and monitoring, in months, after the primary diagnosis. Patients with multiple infection (HIV/EBV-1/2) initiated treatment later. *p˂0.05. (**B**) Evaluation of behavioral profiles at different initiation times for HIV-1 infection treatment and monitoring. Condom use after diagnosis was the most frequent behavior at all treatment initiation times.

In an intergroup analysis, we observed that a time of HIV-1 infection greater than 12 months was more frequent in patients coinfected with HIV/EBV-1/2 (40%; p = 0.0017) and that the chance of multiple infections was approximately 5 times higher (OR: 4.76; 95% CI 1.51–15.04). Most patients in the HIV and HIV/EBV-2 groups had a recent infection diagnosis (< 1 month) (49% and 80%, respectively). A time of infection between 1 and 6 months (78%) prevailed among HIV/EBV-1 coinfected patients, but without statistical significance (Fig. 2A).

We evaluated the behavior of patients based on the time of HIV-1 infection since diagnosis (Fig. 2B). We did not observe significant differences in behavioral profiles; however, regarding sexual practice, we noted an increase in contact with sex workers for patients living longer with the infection; in contrast, the frequency of condom use after diagnosis, an active sex life, and the presence of a steady partner decreased. Regarding nonsexual behavior, alcohol consumption was a constant practice among patients, but we observed a gradual decline in the use of illicit drugs. Additionally, we observed an increase in the frequency of smokers only among patients diagnosed between 7 and 12 months prior.

**Figure 3:**
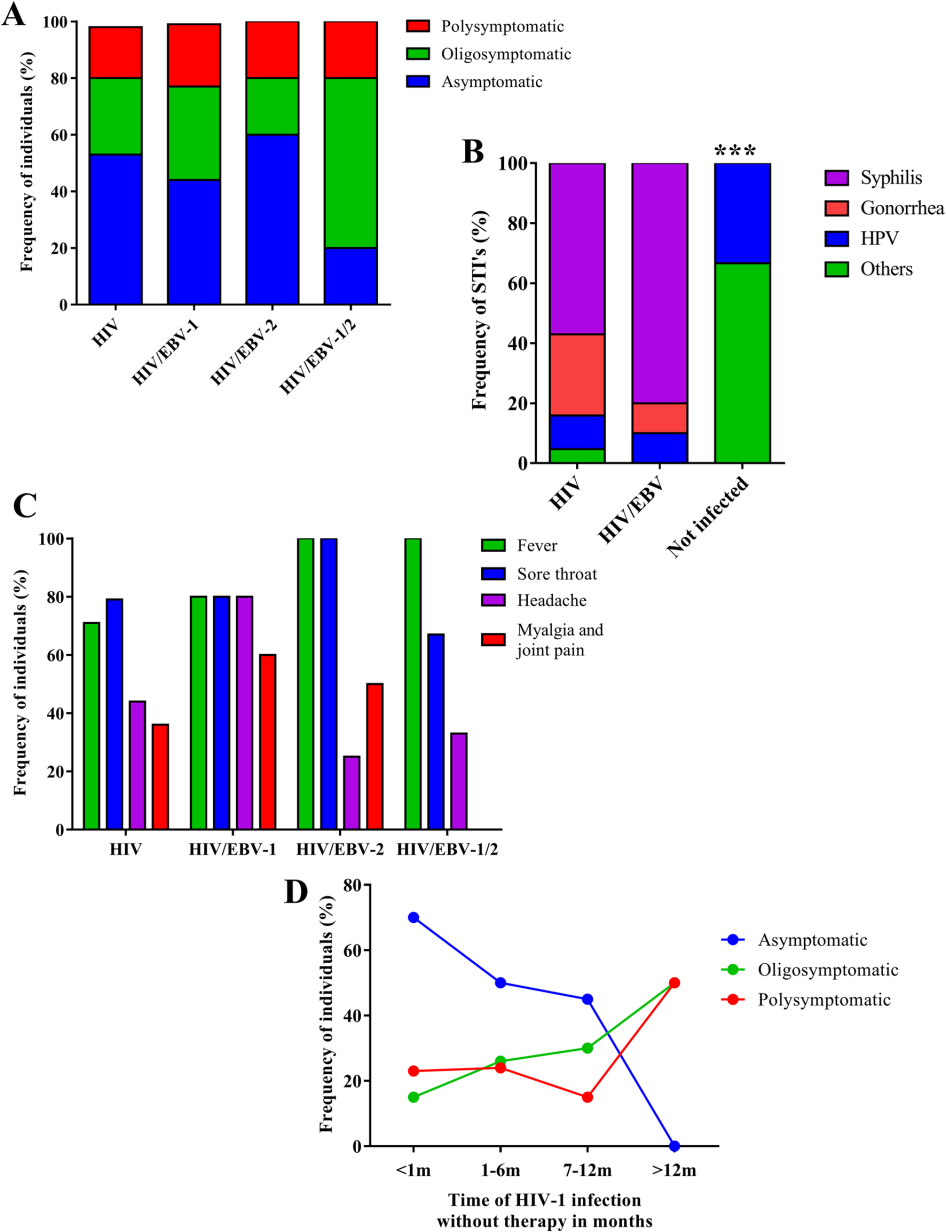
Symptoms: (**A**) Frequency of symptomatological categories among the studied groups. (**B**) STI diversity among individuals with a history of sexually transmitted infections in the studied groups. Blood donors had a poorly diversified history. *** ***: p˂0.001. (**C**) Diversity of symptoms between the studied groups. Fever and sore throat were the most frequent. (**D**) Evaluation of the frequency of the symptomatological categories at different initiation times for HIV-1 infection treatment. There was an extreme reduction in the frequency of asymptomatic patients among patients for whom treatment was initiated greater than 12 months after diagnosis.

We asked the patients about the symptoms presented at the time of collection and grouped them as asymptomatic (without symptoms), oligosymptomatic (from 2 to 3 symptoms) and polysymptomatic (from 4 to 5 symptoms). Asymptomatic patients prevailed in our analyses (51.91%); only in the group of patients coinfected with HIV/EBV-1/2 were oligosymptomatic patients predominant (60.0%), but the difference was not statistically significant (p = 0.96) (Fig. 3A). Sore throat and fever were the most frequent symptoms in all groups (77.78% and 71.72%, respectively), muscle and joint pain were not reported in the HIV/EBV-1/2 group (Fig. 3C), and no significant prevalence of symptoms was observed in the intergroup analysis (p = 0.96).

We evaluated the profile of STIs among individuals with a history of these infections. The occurrence of syphilis and gonorrhea were the most frequent reports in the monoinfected and coinfected groups. The profile of STIs of noninfected individuals was significantly different from that of the other groups (p < 0.0001), with other STIs (66.67%), such as herpes (33.35%) and HPV infection (33.33%), prevailing; candidiasis was an alleged condition in 33.5% of the control group (Fig. 3B).

We evaluated the frequency of the symptomatological groups based on the time since HIV-1 diagnosis (Fig. 3D). We observed a significant decrease in the frequency of asymptomatic patients with an increase in the time since diagnosis (p = 0.04). Conversely, the rate of oligosymptomatic and polysymptomatic patients increased among patients living with HIV-1 for longer.

**Figure 4:**
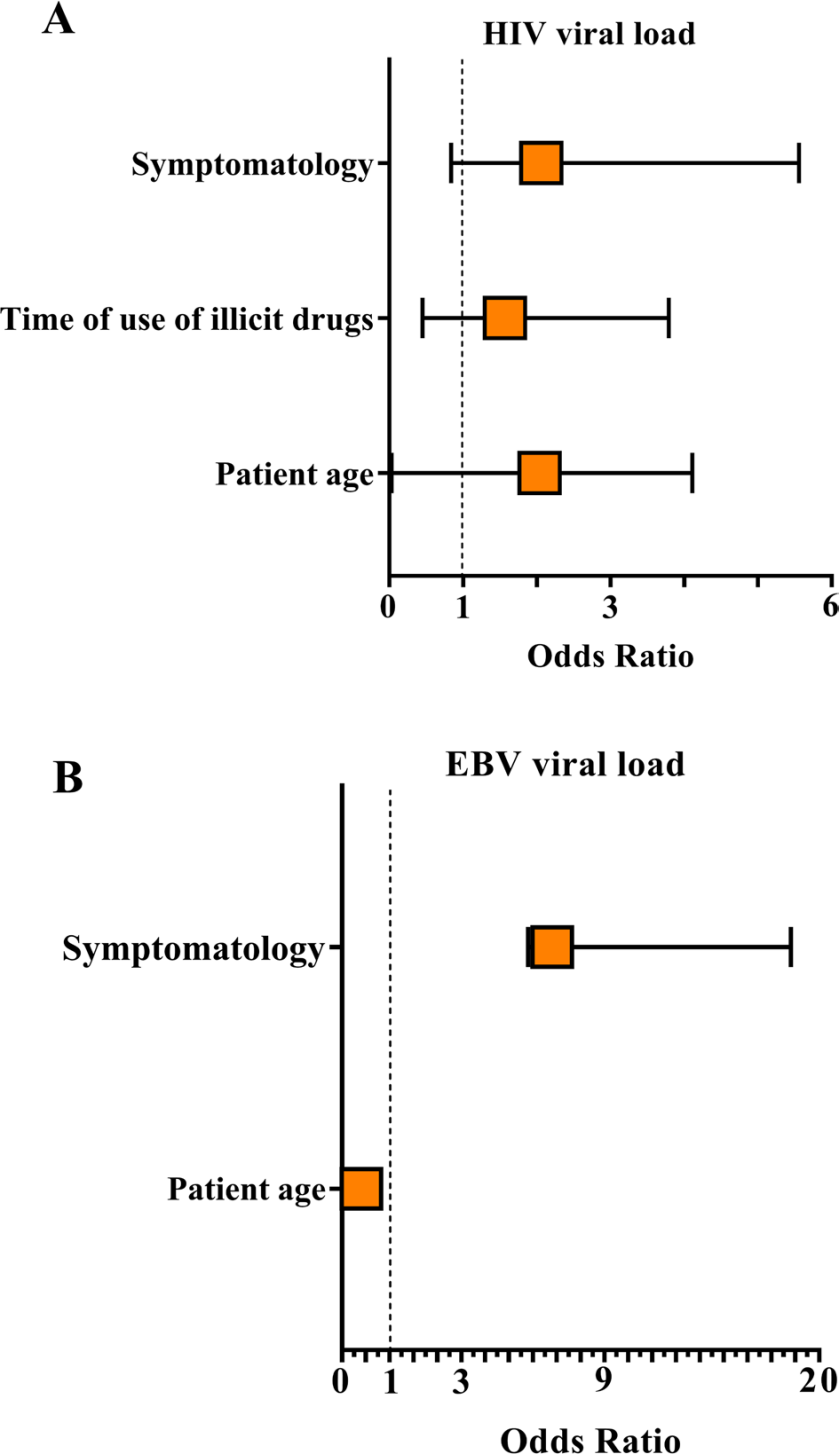
Epidemiological factors associated with increased viral loads: (**A**) epidemiological factors associated with increased HIV viral load. (**B**) Epidemiological factors associated with increased EBV viral load. The x-axis is the odds ratio (OR) values. Horizontal lines represent the 95% confidence interval (CI) for each OR value. Vertical dashed lines delimit an OR equal to 1; therefore, OR values greater than 1 were considered risk factors for increased viral loads. For EBV, when the age of the patients obtained an OR value less than 1, the increase in viral load was inversely proportional to the factor. Symptomatology was a factor dependent on the viral load.

Increased HIV viral load was associated with patient age at the time of collection (OR: 2.04; 95% CI 2.01–2.07; p = 0.026) and with the length of illicit drug use in individuals with an illicit drug use history (OR: 1.57; 95% CI 1.12–2.22; p = 0.0548) (Fig. 4A). For EBV infection, no significant association was observed between viral load and patient’s age, although there seems to be a tendency for older age to be associated with lower viral load values (OR: 0.82; 95% CI 0.79–1.03; p = 0.0579) (Fig. 4B). The replication of both viruses was associated with patient symptoms, especially the EBV viral load, which was approximately 9 times higher in polysymptomatic patients (HIV = OR: 2.06; 95% CI 1.22–3.50; p = 0.0073; EBV = OR: 8.81; 95% CI 1–10; p = 0.0447).

## Discussion

In this study, we identified a prevalence rate of approximately 7% of primary EBV infection in adult HAART-free PLHIV in a state of the Brazilian Amazon region. Our findings were similar to the epidemiological data on the general prevalence of primary infection among immunocompetent adults in emerging countries^35–37^. However, there were discrepancies when compared to data from other studies with patients immunocompromised by HIV-1; our findings were lower than the values observed even in countries with similar socioeconomic profiles^26,38,39^.

Most of the differences between studies are due to the method used for sample screening, which is based on the detection of anti-VCA or anti-Epstein–Barr virus nuclear antigen (EBNA) as markers of EBV infection. The serological panel adopted in the present study was based on the detection of anti-VCA IgM and IgG antibodies, for which we assumed the profile IgM (+) IgG (−) as suspected primary infection, which was confirmed by molecular biology. Although the detection of anti-EBNA IgG antibodies is also suggested as a parameter to distinguish the infectious phases of the disease^40^, methods similar to those used in the present study were indicated for the recognition of primary infection by EBV, with an estimated sensitivity above 98%, and applied in the discrimination of false positive cases^41^. We emphasize that serum anti-VCA IgM levels emerge early and reach their serological peak within the first 5 days of disease onset, a period in which there is an increase in the clinical severity and viral load of EBV in the oral mucosa and peripheral blood^42^. Therefore, we suggest that the panel used in the present study may be a clinically relevant method in the identification of primary and recent EBV infection.

In Brazil, most studies have focused on the detection of EBV in specific organ sites of PLHIV with particular clinical profiles^31,43,44^. The methodologically closest study did not detect the same clinical profile in patients on HAART in southeastern Brazil^45^. We emphasize, however, that in the relatively time period included in the present study, the coinfection rate increased approximately sevenfold per month of collection, while the rate of new cases of PLHIV, understood as therapy-naïve based on the continuous care cascade recommended by the Brazilian Ministry of Health^46^, tended to be stable, with a nonsignificant increase of twofold per month of collection.

Although the number of new HIV cases identified is stable nationally, especially in the northern region of the country^47,48^, our data warn of an increase in coinfections in PLHIV, a predisposition observed in Brazil^49^ and internationally^50^, especially among young men.

We observed a prevalence of EBV-1 among HIV/EBV coinfected patients, confirming the epidemiology of the herpesvirus analyzed in different organ tissues of PLHIV with different clinical profiles^51,52^. Reports of EBV-2 prevalence in coinfected patients occur in specific clinical conditions after transient reactivation events of cell sites infected by different types of EBV^53^.

There is controversy regarding the predominant epidemiological profile among HIV/EBV-1 coinfected patients. Reports indicate that male sex, Caucasian ethnicity and heterosexual preference are factors that influence the distribution of cases^54–56^. In the present study, male individuals predominated, as well as a singular representation of homosexual and bisexual orientation, and we did not evaluate ethnic markers, such as self-reported race. Therefore, we assume a multiethnic representation in our sample with a probable predominance of European ancestry, as already described for populations in the Brazilian Amazon^57,58^. HIV/EBV-2 coinfection combined with multiple infection by both genotypes accounted for more than half the number of coinfections, a finding that emphasizes the susceptibility to the less frequent EBV genotype or to superinfection in HIV-immunocompromised patients^59,60^. It is argued that this is due both to changes in the immunological profile^61^ and in the exposure behavior of the host^62^.

The epidemiological analysis revealed similarities between the monoinfected and coinfected profiles. Apparently, in the context of HIV coinfection, the presence of primary EBV infection is not associated with specific risk factors. In fact, both infections have points of convergence related mainly to unprotected sexual exposure and possible contact with contaminated blood products^63–66^. In the present study, we found a prevalence of factors that are directly and indirectly related to sexual exposure, which makes this the main route of coinfection in the studied context.

The predominant socioeconomic profile was young adult men with a low education level, low family income, and history of illicit drug use. Unsatisfactory education is a risk factor congruent with HIV infection due to the lack of aggregate knowledge about prevention mechanisms of STIs and unprotected sexual exposure^67,68^. When functional, education empowers individuals to both reflect on the extent of contact between people and improves their ability to understand and act in favor of preventive health^69,70^.

It is argued that the lifestyle associated with a high education level can lead to both an increase and a decrease in the risk of HIV infection, depending on the balance of the different influences on behavior^71^. In the present study, more vulnerable socioeconomic profiles were associated with the risk of infection; in contrast, favorable profiles were prevalent among blood donors, a feature relevant to this altruistic activity^72^. This finding illustrates the sociodemographic complexity between the factors 'education' and 'income' that result in better conditions of access and social participation. That in Brazil they may be closely related to the way in which the individual is inserted in the community social organization and is motivated to act and support their relationships^73^. In contrast, low socioeconomic status contributes to less health knowledge and awareness of blood donation needs^74^.

It is suggested that most of the factors that predispose an individual to HIV are associated with family or individual income because this creates environmental risks inherent to precarious access to health, the practice of “sex for survival” (sex as a means of earning income), and even the ability to deal with the consequences of the condition^75,76^. In a context of progressive poverty, HIV mortality and morbidity rates were predicted by lower incomes^77^. Analogously, there is an aggravation of social vulnerability in PLHIV of specific ethnic groups, women, and those with physical health limitations^78^. In contrast, even in already-infected key populations, a relative increase in income results in improvements in access to basic health care^79^.

A history of illicit drug use was the greatest risk factor for monoinfection or coinfection in the present study, among which drugs taken orally prevailed. Recent studies have shown that an increase in the number of noninjectable drug users (NIDUs) infected by HIV trigger epidemiological surveillance in the Amazon region because although this group does not expose itself, a priori, to parenteral transmission, they may be at the mercy of other risk variables, especially sexual factors^80,81^, as observed in the decrease in condom use among NIDU sex workers, even between serodiscordant partners^82^. A worrying problem is the use of polydrug use combined with unprotected sex with multiple partners^83^, especially in Brazil, where although HIV prevalence rates among drug users have been declining in recent years^48^, they can still vary between 10 and 25-fold higher than that estimated in the general population^84^, and most users do not have basic knowledge about the severity of the infection^85^.

From the point of view of sexual practice, homosexual or bisexual orientation without the presence of a steady partner and occasional condom use were predominant characteristics among monoinfected and coinfected individuals. In fact, men who have sex with men (MSM) correspond to one of the main groups particularly vulnerable to HIV (key populations) and who historically do not have adequate access to health services. Data suggest that approximately 47% of new infections worldwide were associated with key populations and their sexual partners in 2017; in that evaluation, the MSM group represented 57% of new cases in Western Europe and Central and North America^86,87^. However, in developed countries, MSM exposed to high risks report willingness to participate in prophylaxis measures once informed of these prevention efforts^88^.

In developing countries, the high proportion of seropositive MSM who are unaware of their serological status is highly worrying and represents a large gap between basic health care practices and national guidelines^89^. In Brazil, recent reports highlight the high prevalence of HIV among MSM, whose main risk factors include environments of vulnerability; stigma and discrimination; behavioral profiles; sexual practices; and lackluster policies and programs^90,91^. Notably, there is a low rate of satisfactory knowledge on HIV infection observed among Brazilian MSM^92^.

As already mentioned, multiple sex partners is one of the characteristics composing the behavioral profiles of MSM^83,93^, and a history of STIs is one of the consequences of this practice^94^, as suggested in the present study. Recent warnings highlight the low rates of disclosure of serological status among sex partners, which is worrisome given the persistence of viral transmissibility, especially when the majority continues to practice unprotected sex^95^. It is argued that specific social networks may be endorsing this exposure behavior^96^. In the present study, we showed that unprotected sex with multiple partners, particularly for young men, are relevant attributes in the prevalence of monoinfection or coinfection. Notably, most infected individuals had their first sexual contact in preadolescence (10 to 15 years), a trend that has been observed in previous studies^97^.

Regarding protected sex, a recent report points to the existence of accessibility barriers for purchasing condoms, even in developed countries, and the need for a multifaceted approach to overcome these barriers^98^. In Brazil, despite an ongoing policy of free provision of condoms in referral units^99^, there is still a deficit in adherence to condom use, as suggested in the present study. We do not know whether this scenario is due to poor adherence itself, as a behavioral attitude^100^, to stigmatization in accessibility^101^, or to population ignorance regarding the national condom distribution policy. Future studies addressing this issue are essential as a strategic basis for preventive measures in the Brazilian Amazon region.

A history of STIs was the second factor most strongly associated with monoinfection or coinfection, with a prevalence of reported cases of syphilis and gonorrhea, respectively. Recent studies have shown that the highest risk for STIs is observed in young MSM from the most vulnerable socioeconomic groups^50,62^; among these, those diagnosed with syphilis and gonorrhea have the highest rate of HIV coinfection^102^, similar to our findings. These data are particularly worrying for Brazil because the incidence rates of acquired syphilis have been increasing since 2010^103^, and there is an estimated global increase in cases of gonorrhea, especially in MSM in developing countries^104^. The specific detection of these STIs can guide resource-intensive interventions, such as pre-exposure prophylaxis (PrEP), among PLHIV^102^.

In summary, we propose a risk/exposure potential matrix for monoinfection or coinfection based on sexual and social risk factors. We showed that the highest risk/exposure occurs among individuals with social vulnerability and promiscuous sexual behavior, among whom condom nonusers were more susceptible to viral infections.

However, condom use had a risk more associated with low education and low family income, similar to that presented among immigrants with similar socioeconomic profiles. In this scenario of preventive failure, we assume that social factors can directly influence the quality of aggregated knowledge about proper condom handling and use and awareness of STI/AIDS^105^. Alternatively, high education is associated with affordable family income and consequent adoption of safer sexual practices and access to preventive health services^106^, as suggested for individuals with high purchasing power, whose risk/exposure remained modest regardless of sexual factors.

We noted that on a monthly scale, the care and monitoring of HIV infection in a portion of patients was initiated late, with an interval between 1 and 24 months after becoming aware of the infection. However, the majority started the process quickly, approximately 1 month after the primary diagnosis.

These results are promising given persistent reports of late diagnosis among PLHIV. The greatest concern lies with the increase in the degree of morbidity and mortality inherent to the late initiation of treatment, although a trend toward a decrease in overall lethality is observed in these patients, despite maintaining a high frequency of late or advanced presentation of HIV-infected patients and the evolution of associated factors^107^. It is argued that in these cases, the immediate initiation of HAART may have favored the gradual immune recovery of patients and impacted mortality^108^. In Brazil, management guidelines advocate the immediate start of therapeutic regimens regardless of the patient’s clinical or immunological status^46^.

One strategy to encourage the early diagnosis of STIs is to promote testing outside the health setting^109,110^. Brazil again stands out for encouraging this practice in official and independent campaigns that aim not only to diagnosis but also to provide information regarding and encourage condom use^111,112^.

It is understood that older age and heterosexual orientation are the main factors influencing late start of monitoring in PLHIV^113–116^; however, little is discussed about the risk behavior profile of this group. In the present study, we observed that the exposure behavior was similar between the different times since diagnosis, indicating that prolonged experience with HIV did not significantly alter the behavioral profile of patients who maintained promiscuous practices related to infection and inherent comorbidities. Although there was a decrease in the percentage of sexual practice in patients for whom treatments was initiated late, most patients had no steady partner and maintained sexual contact with sex workers, some of whom did not use condoms.

We also show that the risk of HIV/EBV-1/2 multiple infection was higher in patients with longer HIV-1 infection, which emphasizes our warning because coinfections are common in patients who start treatment late, have the highest associated risk rate, and are also correlated with advanced clinical disease stages^117,118^. For EBV, this becomes more specific because the prevalence of EBV-1 and of multiple infection predominate in the most immunosuppressed patients; however, it is not known which factors may be involved in this scenario^44,119^.

Regarding the symptomatological aspects, asymptomatic individuals prevailed in all groups. Symptoms, when present, although still noncomplex and clinically nonspecific, were similar between both the initial phase of HIV infection and primary symptomatic EBV infection^120–123^. Particularly with EBV, these symptoms are associated with age groups above 18 years old and the host's aggravated immune status^64^, attributes that were prevalent among coinfected patients in the present study.

With the late initiation of HAART, there was a clear progression in coinfection symptoms, evidenced by the increase in cases of oligosymptomatic and polysymptomatic patients with HAART initiation greater than 12 months after the primary diagnosis. Although our results indicate that most patients sought specific medical care at the onset of the disease, we show how neglect of the condition can affect the pathological status of HIV infection, given that antiretroviral therapy initiated as soon as possible delays the progression of the disease and viral transmission^124^ and has secondary benefits^125,126^.

We observed that some of the epidemiological and behavioral factors were also associated with biological aspects of coinfection. Exclusively for HIV, patients with longer use of illicit drugs were more likely to have a high viral load. Specifically, we noted that most users used cocaine or marijuana, which are associated with effects on the immune system indicative of compromised resistance to HIV^127^, which favors not only the risk of infection but also intensive therapeutic control^128^. We suggest that the continued use of these illicit drugs may be conducive to both the acquisition and progression of infection because the time of initiation of HAART was not associated with viral load, which points to a complex interaction between illicit drug use and virological activity, which may be as significant as exposure to therapy^129^.

The advancement of age as a risk factor for increased HIV viral load was a finding different from those reported in previous studies^130–132^. However, in the present study, all patients were treatment-naïve, unlike those participating in the aforementioned studies, revealing the possible effects of immunosenescence in the face of HIV infection^133^, even in a predominantly young population.

In contrast, the chances of the EBV viral load increasing were higher in younger patients, in contrast that what was observed in HIV patients. Our findings reflect those observed in other epidemiological studies, in which it is argued that the early acquisition of primary infection results in higher and more sustained levels of EBV viremia. The presence of coinfections may also impair specific immune surveillance for one or both pathogens, leading to worsening of the disease^134,135^.

For both viruses, symptomatology was a factor dependent on viral load; the higher the viral load is, the greater the chances of patients presenting with a polysymptomatic condition, especially in EBV infection. For HIV, in cohorts with a high prevalence of infection, it was shown that viral load is a determining factor of disease progression^136^, an observation more characteristic of the acute phase of infection^123^. However, studies indicate that viral subtype and chronic immune activation are also aspects that predict the course of progression in populations of different ethnic origins^137,138^. For EBV, the plasma viral load is directly proportional to the patient's symptoms, especially within the first 2 weeks of symptom onset; in the acute phase, viral replication is intense in peripheral blood, with the virus contained in infected B cells and a portion being released into the plasma^64,139^. In specific clinical cases, the plasma viral load, even when low, is already an effective biomarker^140,141^.

## Conclusions

Based on the results of the present study, we conclude that the prevalence of primary coinfection with EBV in HIV therapy-naïve patients in a state of the Brazilian Amazon region was lower than that observed in other similar regions. This finding may be associated with the methods used in the different studies, with social vulnerability and promiscuous sexual behavior being the risk factors associated with this population. We warn of the effect of the late initiation of HIV infection monitoring and the consequences of this decision on the risk of coinfections by EBV and disease progression, and we suggest that some epidemiological factors may influence the increase in viral load of both viruses.

## Data Availability

Data supporting the findings of this study are available from Igor Brasil Costa, but restrictions apply to the availability of these data, which were used under license for the present study and therefore are not publicly available. However, data are available from the authors upon reasonable request and with permission from Igor Brasil Costa.
