# Author Correction: Epidemiological risk factors associated with primary infection by Epstein–Barr virus in HIV-1-positive subjects in the Brazilian Amazon region

**DOI:** 10.1038/s41598-022-12511-y

**Published:** 2022-05-17

**Authors:** Leonn Mendes Soares Pereira, Eliane dos Santos França, Iran Barros Costa, Igor Tenório Lima, Amaury Bentes Cunha Freire, Francisco Lúzio de Paula Ramos, Talita Antonia Furtado Monteiro, Olinda Macedo, Rita Catarina Medeiros Sousa, Felipe Bonfim Freitas, Igor Brasil Costa, Antonio Carlos Rosário Vallinoto

**Affiliations:** 1 Laboratory of Virology, Institute of Biological Sciences, Federal University of Pará, Belém, Pará Brazil; 2 Epstein-Barr Virus Laboratory, Virology Section, Evandro Chagas Institute, Ananindeua, Pará Brazil; 3 Department of Epidemiology and Surveillance, Evandro Chagas Institute, Ananindeua, Pará Brazil; 4 Laboratory of Retroviruses, Evandro Chagas Institute, Virology Section, Ananindeua, Pará Brazil; 5 School of Medicine, Federal University of Pará, Belém, Pará Brazil; 6 Graduate Program in Biology of Infectious and Parasitic Agents, Institute of Biological Sciences, Federal University of Pará, Belém, Pará Brazil

Correction to: *Scientific Reports* 10.1038/s41598-021-97707-4, published online 16 September 2021

The original version of this Article contained an error in Figure 1, where Panel B was incorrect and Panel C was published as Panel B. The original Figure 1 and accompanying legend appear below.

**Figure 1:**
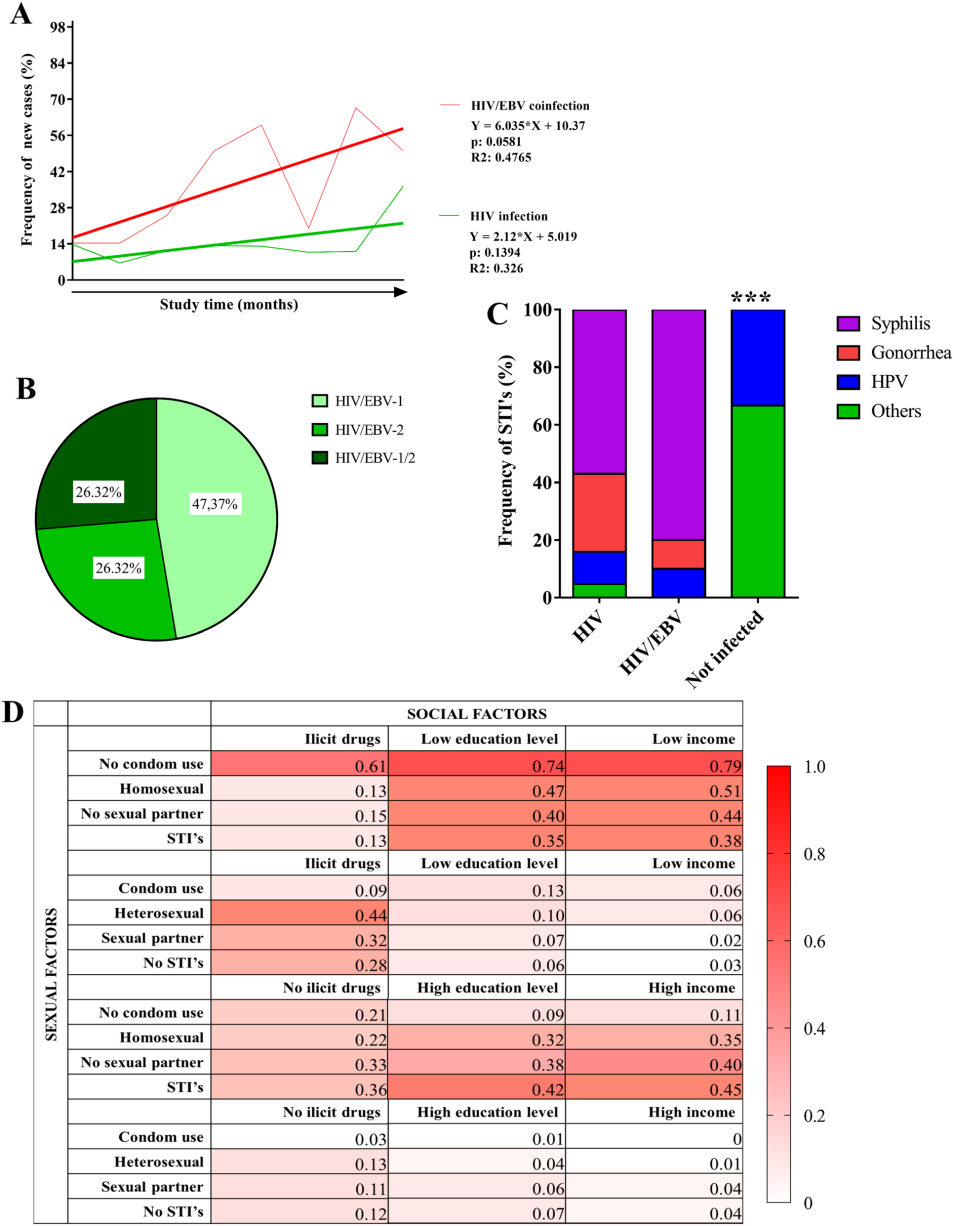
Frequency of cases and degree of exposure: (**A**) Frequency of new cases of HAART-free HIV-1 patients coinfected with HIV/EBV in the period between January 2018 and December 2019. (**B**) Prevalence of cases of HIV/EBV coinfection stratified based on age and sex. (**C**) Prevalence of EBV genotypes among HIV/EBV coinfected patients. (**D**) Potential risk/exposure to HIV-1 monoinfection or co-infection. The color gradient was proposed based on data on the frequency of individuals screened according to the intersection of social and sexual factors.

The original Article has been corrected.

